# Case Report: Right frontal periventricular heterotopia underlying secondary network epilepsy with a Lennox–Gastaut phenotype: multimodal SEEG, CCEP, and rs-fMRI evidence

**DOI:** 10.3389/fnins.2026.1961911

**Published:** 2026-09-30

**Authors:** Changquan Wang, Jing Hong, Jialin Ji, Caicai Zhang, Wenzhen Chen, Jiwen Xu

**Affiliations:** 1 Department of Functional Neurosurgery, Comprehensive Epilepsy Unit, Ruijin Hospital Luwan Branch, Shanghai Jiao Tong University, School of Medicine, Shanghai, China; 2 Department of Neurosurgery, Clinical Neuroscience Center, Comprehensive Epilepsy Unit, Ruijin Hospital, Shanghai Jiao Tong University School of Medicine, Shanghai, China; 3 Department of Radiology, Ruijin Hospital Luwan Branch, Shanghai Jiao Tong University, School of Medicine, Shanghai, China

**Keywords:** cortico-cortical evoked potentials, Lennox–Gastaut phenotype, periventricular heterotopia, resting-state functional MRI, stereo-electroencephalography

## Abstract

**Objective:**

Lennox–Gastaut syndrome (LGS) is generally regarded as a developmental and epileptic encephalopathy involving distributed brain networks, although a focal structural abnormality may occasionally recruit bilateral cortical and thalamic circuits and produce an LGS phenotype. We characterized the spatiotemporal organization of such a network and its changes after focal SEEG-guided radiofrequency thermocoagulation (RF-TC).

**Methods:**

A 50-year-old woman with drug-resistant epilepsy, multiple seizure types, diffuse slow spike-and-wave discharges, generalized paroxysmal fast activity, intellectual impairment, and right frontal periventricular heterotopia (PVH) underwent stereo-electroencephalography (SEEG). Cortico-cortical evoked potentials (CCEPs) were acquired before and after SEEG-guided radiofrequency thermocoagulation (RF-TC). Resting-state functional MRI (rs-fMRI), obtained before and 3 months after RF-TC, was analyzed using three seeds: the entire right frontal PVH and the right and left centromedian thalamic nuclei (CM).

**Results:**

SEEG identified the earliest sustained ictal activity within the sampled right frontal PVH-related medial contacts, accompanied by high-frequency activity. This was followed by ipsilateral lateral frontal recruitment, contralateral frontal synchronization, and subsequent bilateral CM involvement. Two-stage RF-TC of the onset and early-recruited networks was followed by seizure freedom through 9 months. After RF-TC, right-sided stimulation produced markedly attenuated lesion-related CCEP responses, whereas left-sided responses and responses between thalamic contacts showed little appreciable change. Descriptive rs-fMRI difference maps demonstrated decreased local frontal connectivity around the treated territory and increased long-range connectivity for the PVH seed; both CM seeds showed predominantly increased connectivity with distributed cortical and cerebellar regions.

**Conclusion:**

In this patient, a focal PVH-related epileptogenic network was associated with secondary bilateral cortical and thalamic recruitment underlying an LGS phenotype. CCEP and rs-fMRI captured complementary aspects of postoperative network change: attenuation of stimulation-evoked propagation and postoperative changes in intrinsic functional connectivity, respectively.

## 1 Introduction

Lennox–Gastaut syndrome (LGS) is a severe developmental and epileptic encephalopathy defined by multiple seizure types, diffuse slow spike-and-wave (SSW) and generalized paroxysmal fast activity (GPFA) on EEG, and intellectual disability (Arzimanoglou et al., 2009; Specchio et al., 2022). Although conventionally classified as a generalized epilepsy syndrome, electrophysiological and imaging data support a distributed network model in which focal lesions can recruit bilateral cortical and thalamocortical circuits (Archer et al., 2014; Warren et al., 2019; Cho et al., 2025). Archer et al. proposed that LGS represents “secondary network epilepsy,” in which a focal or multifocal lesion destabilizes large-scale networks (Archer et al., 2014). Warren et al. further showed that GPFA is often cortically driven, with early prefrontal involvement followed by brainstem and thalamic engagement (Warren et al., 2019). Intracranial data identify bilateral fronto-motor-parietal networks as core nodes, with neocortex as a critical generator (Cho et al., 2025). The centromedian thalamic nucleus (CM) is strongly connected with widespread cortical regions and is an established neuromodulation target in LGS (Dalic et al., 2021). Its position within the ictal hierarchy, however, may vary among patients. Scalp EEG and functional imaging cannot reliably determine whether thalamic activity initiates a seizure, participates in its early organization, or follows cortical recruitment. Stereo-electroencephalography (SEEG) can resolve this temporal relationship, while cortico-cortical evoked potentials (CCEPs) and resting-state functional MRI (rs-fMRI) provide complementary measures of stimulation-evoked and intrinsic connectivity.

Direct electrophysiological evidence linking focal cortical or heterotopic lesions to bilateral synchrony and thalamic engagement remains limited. We report a patient with a right frontal periventricular heterotopia (PVH) and an LGS phenotype who underwent SEEG-guided radiofrequency thermocoagulation (RF-TC). We sought to define the ictal hierarchy, assess peri-interventional changes in effective propagation using CCEPs, and describe longitudinal intrinsic functional connectivity using three anatomically defined rs-fMRI seeds: the PVH and bilateral CM nuclei.

## 2 Materials and methods

### 2.1 Patient and presurgical evaluation

A 50-year-old right-handed woman was referred for evaluation of long-standing drug-resistant epilepsy. Early development had been normal, and seizures began at 10 years of age. She subsequently discontinued formal education and had no formal occupation. Despite multiple antiseizure medication regimens, seizures remained refractory. Seizure-related morbidity included severe hot-water burns requiring skin grafting and frontal skull deformity caused by repeated falls. At admission, she was taking levetiracetam 1 g twice daily and oxcarbazepine 0.45 g twice daily.

Neuropsychological assessment showed mild intellectual disability (full-scale IQ, 65), moderate memory impairment (memory quotient, 39), and impaired executive function and processing speed (Montreal Cognitive Assessment score, 15). Structural MRI demonstrated a gray-matter-signal abnormality adjacent to the frontal horn of the right lateral ventricle and extending radially toward the lateral frontal cortex, consistent with PVH. 18F-fluorodeoxyglucose PET showed corresponding right frontal hypermetabolism.

Video-EEG captured tonic seizures, epileptic spasms, spasm–tonic seizures, and one focal-to-bilateral tonic–clonic seizure. Interictal EEG demonstrated diffuse 1.5–2.5-Hz SSW discharges, and GPFA accompanied tonic seizures (Supplementary Figure S1). The clinical history, seizure types, EEG features, and cognitive impairment supported drug-resistant epilepsy with an LGS phenotype.

### 2.2 SEEG implantation and recording

SEEG was undertaken to clarify the relationship between the focal right frontal abnormality and the generalized electroclinical phenotype. The implantation strategy addressed three questions: whether seizures originated within the right frontal lesional/perilesional network, whether contralateral frontal activity represented propagation, and when the bilateral CM nuclei were recruited relative to cortical seizure onset.

Five right frontal electrodes (A–E) sampled the lesional and perilesional network. Electrodes A, B, C, and E traversed the right frontal PVH, whereas electrode D targeted the region posterior to the lesion. To assess contralateral involvement and interhemispheric propagation, electrode A’ was positioned in the contralateral frontal cortex. Electrodes R and L’ targeted the right and left CM nuclei, respectively, to assess thalamic recruitment relative to cortical seizure onset (Figure 1).

**Figure 1:**
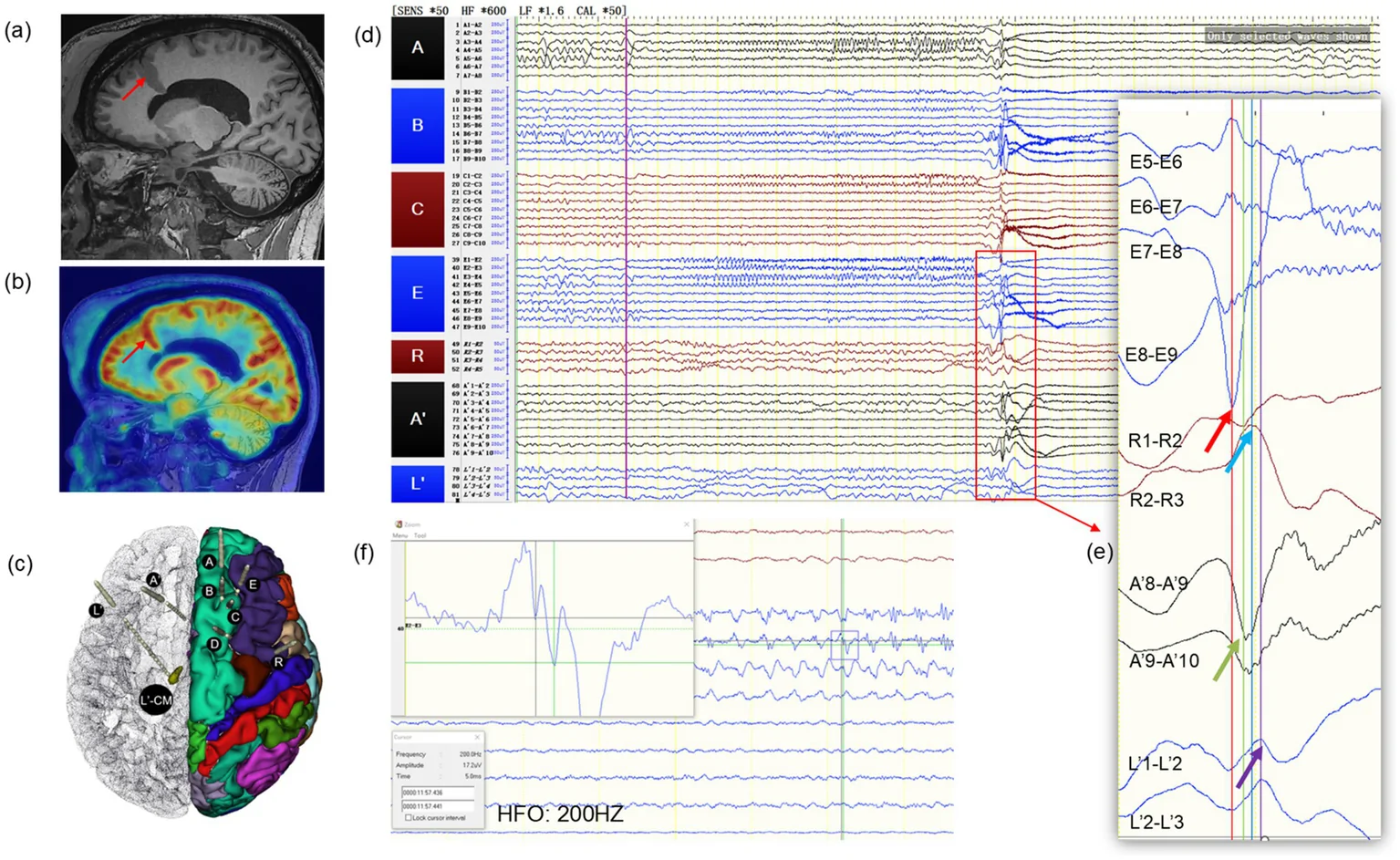
Presurgical localization and SEEG-defined spatiotemporal organization of the right frontal PVH-related epileptic network. **(a)** MR sagittal view showing the periventricular PVH-related region (red arrow). **(b)** PET sagittal view demonstrating hypermetabolic activity corresponding to the PVH-related area (red arrow). **(c)** Three-dimensional reconstruction shows SEEG coverage of the right frontal lesional/perilesional network (A–E), contralateral frontal cortex (A’), and bilateral centromedian nuclei (right CM, R; left CM, L’). **(d)** The purple vertical line indicates the earliest sustained ictal activity detected in the medial PVH-related contacts (E1–4, C1–3, A1–5, and B1–3). The approximately 5.6-s interval from medial ictal onset to the emergence of large-amplitude ictal waveforms in the right lateral frontal cortex is indicated. The red box highlights the subsequent rapid propagation sequence, shown in an expanded view in panel **(e)**. **(e)** Expanded recording of the rapid propagation sequence following recruitment of the right lateral frontal cortex. Vertical markers indicate recruitment in the right lateral frontal cortical contacts of electrode E (red), contralateral frontal electrode A’ (green), right CM electrode R (blue), and left CM electrode L’ (purple). Recruitment occurred from E to A’ after 19 ms, from E to right CM after 30 ms, and from right CM to left CM after a further 12 ms. **(f)** Visual morphology-based analysis of contact E2 demonstrates early high-frequency activity centered at approximately 200 Hz. Lowercase letters denote figure panels; uppercase letters denote SEEG electrode groups. CM, centromedian thalamic nucleus; PVH, periventricular heterotopia.

High-frequency activity (HFA) was identified by two experienced epileptologists through visual analysis of SEEG recordings acquired using a Nihon Kohden recording system with a 256-channel amplifier (Nihon Kohden Corporation, Tokyo, Japan) at a sampling rate of 5,120 Hz. After exclusion of white-matter contacts, recordings were reviewed with eight channels displayed per 1-s page. Signals were high-pass filtered at 80 Hz, and oscillation frequency was measured using the software’s built-in measurement tool.

Activity was classified as genuine HFA only when the oscillatory pattern was clearly visible in the unfiltered raw signal and the peaks and troughs of the filtered trace were temporally aligned with those in the raw signal. Filter-related ringing and other artifacts were excluded when the oscillations were absent from the raw signal or lacked consistent temporal alignment. The identified activity was centered at approximately 200 Hz and fell within the ripple range (80–250 Hz). Because this was a visual, morphology-based analysis rather than a quantitative time–frequency power analysis, no baseline correction or normalization was performed.

### 2.3 Cortico-cortical evoked potentials (CCEPs): stimulation and analysis

CCEPs were acquired before and after RF-TC under comparable awake conditions and with an unchanged antiseizure medication regimen. Post-RF-TC CCEP was performed 4 days after the second-stage RF-TC. SEEG signals were recorded using a bipolar montage between adjacent contacts on the same electrode. Identical stimulation and recording pairs, stimulation parameters, and acquisition settings were used in both sessions. Bipolar stimulation consisted of biphasic square-wave pulses (0.3-ms pulse width) delivered at 1 Hz and 2 mA for 60 stimuli. Intracranial EEG was continuously monitored for after-discharges, and stimulation was discontinued if epileptiform after-discharges or clinical seizures occurred.

For each stimulation pair, the stimulated contacts were excluded from recording analyses. Signals were band-pass filtered (0.5–30 Hz), epoched from −0.2 to 0.8 s, and baseline-corrected using the −200 to 0 ms interval. Data within ±5 ms of stimulus onset were set to zero to reduce stimulation artifacts. Trials with significant noise or artifacts, including those recorded during or after clinical seizures, were excluded by visual inspection. Stimulation pairs A4–A5, A5–A6, and A6–A7 were excluded from both sessions because of severe 50-Hz noise.

Remaining trials were averaged for each stimulation–recording pair. CCEP response strength was quantified as the RMS amplitude from 15 to 300 ms after stimulation. Median RMS values across recording contacts and percentage reductions, calculated as (Pre − Post) / Pre × 100% were summarized separately for the right and left hemispheres using medians and IQRs.

### 2.4 Resting-state functional MRI data acquisition and analysis

Resting-state functional MRI (rs-fMRI) data were acquired before and after SEEG-guided RF-TC 3 months later. Single-shot echo-planar BOLD fMRI: TR 700 ms, TE 30 ms, flip angle 52°, 63 contiguous slices (2.5 mm), 2.5 mm isotropic voxels, 700 volumes (~8 min). Antiseizure medications were unchanged.

Preprocessing in DPARSFA 5.5 included removal of the first 10 volumes, slice-timing and motion correction, T1 co-registration, segmentation, nuisance regression (white matter, CSF, motion), MNI-152 normalization (2 mm), band-pass filtering (0.01–0.1 Hz), and 4 mm FWHM smoothing.

Three seed regions were analyzed: (1) the entire right frontal PVH, (2) the right CM, and (3) the left CM. The PVH was manually delineated on the individual T1-weighted image according to its visible anatomical boundaries and transformed into MNI space. The CM regions were obtained from FreeSurfer automated thalamic nuclei segmentation of the individual structural image. Seed-to-seed correlation coefficients were Fisher r-to-z transformed. For visual description of longitudinal change, postoperative-minus-preoperative seed-to-voxel maps were displayed using the top 5% of difference-map intensity and a minimum cluster size of 200 voxels. These display thresholds were exploratory and were not treated as inferential statistical significance thresholds.

## 3 Results

The timeline of clinical evaluation and intervention is summarized in Table 1.

**Table 1:** Timeline of clinical evaluation and intervention.

Time point	Event	Description
Day 0 + 11	Baseline evaluation	Scalp video-EEG, structural MRI, Resting-state fMRI, FDG-PET, neuropsychological assessment
Day 12	SEEG implantation	8 electrodes implanted
Day 13 + 4	SEEG recording	No seizure
Day 17+	SEEG recording	Habitual seizures captured
Day 23	SEEG recording	Pre-op CCEP
Day 24	Stage 1 RF-TC	Target: A1–5, B1–3, C1–3, E1–4
Day 27	Stage 2 RF-TC	Target: B6–10, C5–10, E5–9
Day 31	Post-op SEEG/CCEP	Repeat CCEP recording to assess effective connectivity changes
Day 32	SEEG electrode explantation	SEEG electrodes removed after completion of CCEP recording
Day 33	Discharge	Patient discharged in stable condition
3 months post-op	rs-fMRI follow-up	Resting-state fMRI acquired
6 months post-op	Clinical follow-up	Engel I (seizure-free); ASMs unchanged
9 months post-op	Clinical follow-up	Engel I (seizure-free); ASMs unchanged

EEG, electroencephalography; FDG-PET, fluorodeoxyglucose positron emission tomography; MRI, magnetic resonance imaging; RF-TC, radiofrequency thermocoagulation; SEEG, stereoelectroencephalography; CCEP, cortico-cortical evoked potentials; rs-fMRI, resting-state functional MRI; ASMs, antiseizure medications.

### 3.1 SEEG defined a right frontal PVH-related ictal hierarchy

Multiple habitual seizures were captured during SEEG monitoring. Despite generalized scalp EEG features, SEEG demonstrated a right frontal lesion-related epileptic network with secondary bilateral cortical and thalamic involvement. Notably, no seizures occurred during the first 4 days after electrode implantation, despite a preimplantation seizure frequency of dozens of spasms or tonic seizures per day.

Two ictal electrographic patterns were identified. The principal pattern included 10 spasm-tonic seizures and one evolving into a focal-to-bilateral tonic–clonic seizure. All 11 seizures showed a consistent spatiotemporal hierarchy despite variations in initial electrographic morphology. Earliest ictal activity (within sampled medial PVH-related contacts) was defined as the earliest sustained ictal activity detected in the medial PVH-related contacts (E1–4, C1–3, A1–5, and B1–3). Prominent large-amplitude ictal waveforms emerged in the right lateral frontal cortex approximately 5.6 s later. Only after this lateral recruitment did activity propagate to the contralateral lateral frontal cortex (19 ms), right centromedian nucleus (CM; 30 ms), and subsequently the left CM (a further 12 ms). Visual analysis of the raw and high-pass-filtered SEEG recordings identified reproducible high-frequency activity centered at approximately 200 Hz in the medial contacts, supporting their early involvement (Figure 1). In the second electrographic pattern, lateral frontal slow-wave/sharp-wave activity preceded attenuation and high-frequency recruitment, but high-frequency activity emerged earlier in medial lesion-related than lateral contacts (Supplementary Figure S2). Thus, generalized scalp features reflected secondary bilateral cortical and thalamic recruitment from a right frontal PVH-related epileptogenic core. Seizure counts and hierarchy reproducibility are summarized in Supplementary Table 1.

### 3.2 SEEG-guided staged RF-TC and immediate treatment response

The two RF-TC stages were separated by 3 days. Bipolar RF-TC was performed between adjacent contacts of the target electrodes at 6 W for 30 s. After the first stage, which targeted the medial seizure-onset network (E1–4, C1–3, A1–5, and B1–3), no seizures occurred during the interval before the second stage. Activity at the RF-TC-treated contacts became low voltage, although focal fast activity persisted at B7–10, C8–10, E6–9. The second stage targeted B6–10, C5–10, and E5–9. Thereafter, seizure freedom was sustained, and the residual fast activity was markedly reduced in amplitude and frequency, with focal slow-wave activity observed. The patient experienced mild headache and a transient low-grade fever of approximately 37.8 °C. No hemorrhage, infection, or new neurological deficit occurred after either stage.

### 3.3 CCEP demonstrated disruption of the right frontal-driven network after RF-TC

Before RF-TC, CCEP mapping from all right-sided stimulation sites revealed widespread bilateral responses across the brain. After RF-TC, the response matrix showed a marked reduction in responses, with only a limited number of high-amplitude responses remaining (Figure 2). In contrast, left-sided stimulation elicited relatively sparse and localized responses, with little postoperative change (Supplementary Figure S3). Despite this overall attenuation, responses recorded from the CM were relatively preserved.

**Figure 2:**
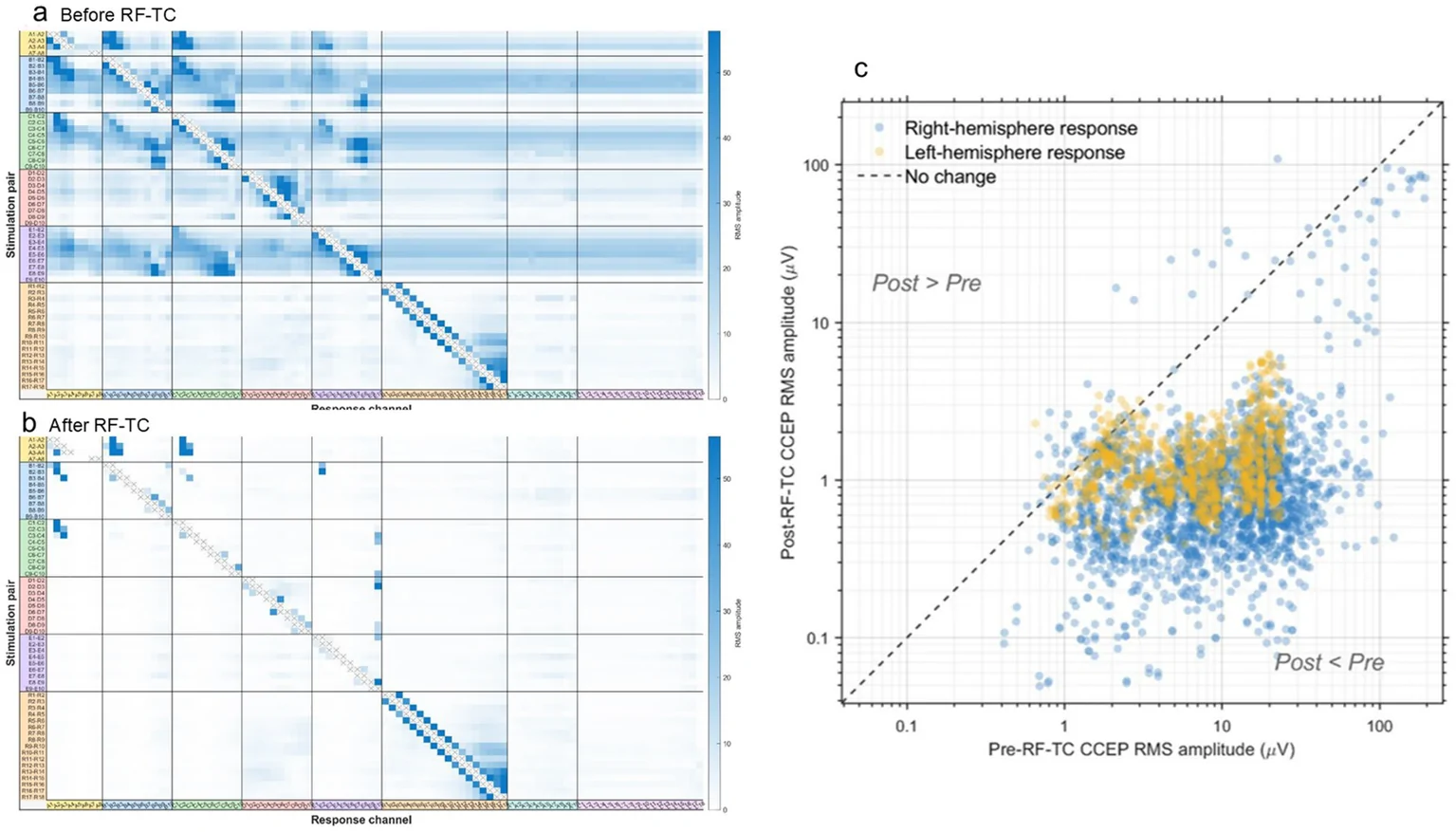
CCEP responses to right-sided stimulation before and after RF-TC. Heatmaps show CCEP responses before **(a)** and after **(b)** RF-TC. RF-TC was performed only in the right hemisphere. In **(a,b)**, rows represent all right-sided bipolar stimulation pairs, and columns represent response channels from both hemispheres. Response strength was quantified as the RMS amplitude from 15 to 300 ms after stimulus onset. Crossed gray cells correspond to contact pairs involved in the stimulation and were excluded from the recording analysis. Darker blue indicates stronger responses. **(c)** Pre–post changes in whole-brain CCEP responses to stimulation involving the ablated region. Only stimulation pairs with at least one contact located within the ablated region were included. Each point represents one stimulation–response contact pair. The *x*- and *y*-axes show the CCEP RMS amplitude before and after RF-TC, respectively. Blue and yellow indicate responses recorded from the right and left hemispheres, respectively. The dashed diagonal line represents (*y* = *x*); points below and above the line indicate decreased and increased CCEP amplitude after RF-TC, respectively.

Quantitative analysis included 35 right-sided stimulation pairs with at least one contact within the ablation zone. For each stimulation pair, the RMS amplitude was summarized as the median across all recording contacts. The median RMS amplitude decreased from 8.88 μV (IQR, 4.03–15.49) before RF-TC to 0.87 μV (IQR, 0.79–1.12) after RF-TC. The median relative reduction across stimulation pairs was 90.4% (IQR, 79.2–92.6%). Among 3,220 matched stimulation–recording contact pairs, 3,133 (97.3%) showed lower RMS amplitudes after RF-TC. When stratified by recording hemisphere, reductions occurred in 2,185 of 2,240 right-hemisphere pairs (97.5%) and 948 of 980 left-hemisphere pairs (96.7%), with median pairwise relative reductions of 91.5 and 83.6%, respectively (Figure 2).

### 3.4 Postoperative changes in intrinsic connectivity after RF-TC

Motion-quality measures were comparable between the two rs-fMRI scans. Mean framewise displacement was 0.146 mm preoperatively and 0.147 mm postoperatively, and maximum absolute translation was below 1 mm in both scans. No volume exceeded an FD of 0.5 mm in the preoperative scan, whereas three volumes (0.4%) exceeded this threshold in the postoperative scan. ROI-to-ROI analysis showed increased postoperative functional connectivity between the PVH seed and bilateral CM nuclei, as well as between the CM nuclei (Supplementary Table S2). In seed-to-voxel analyses, the PVH seed showed decreased connectivity in ipsilateral frontal regions surrounding the PVH and RF-TC territory, including superior/middle frontal and anterior midline areas, and increased connectivity in posterior midline, supplementary motor, and cerebellar regions. Right and left CM seeds showed predominantly increased connectivity with distributed cortical and cerebellar territories, with few negative clusters (Figure 3). These single-subject pre–post findings are descriptive and exploratory. Postoperative changes within the PVH seed itself may have influenced the observed connectivity differences. Therefore, these results cannot be taken as evidence of treatment-induced network changes.

**Figure 3:**
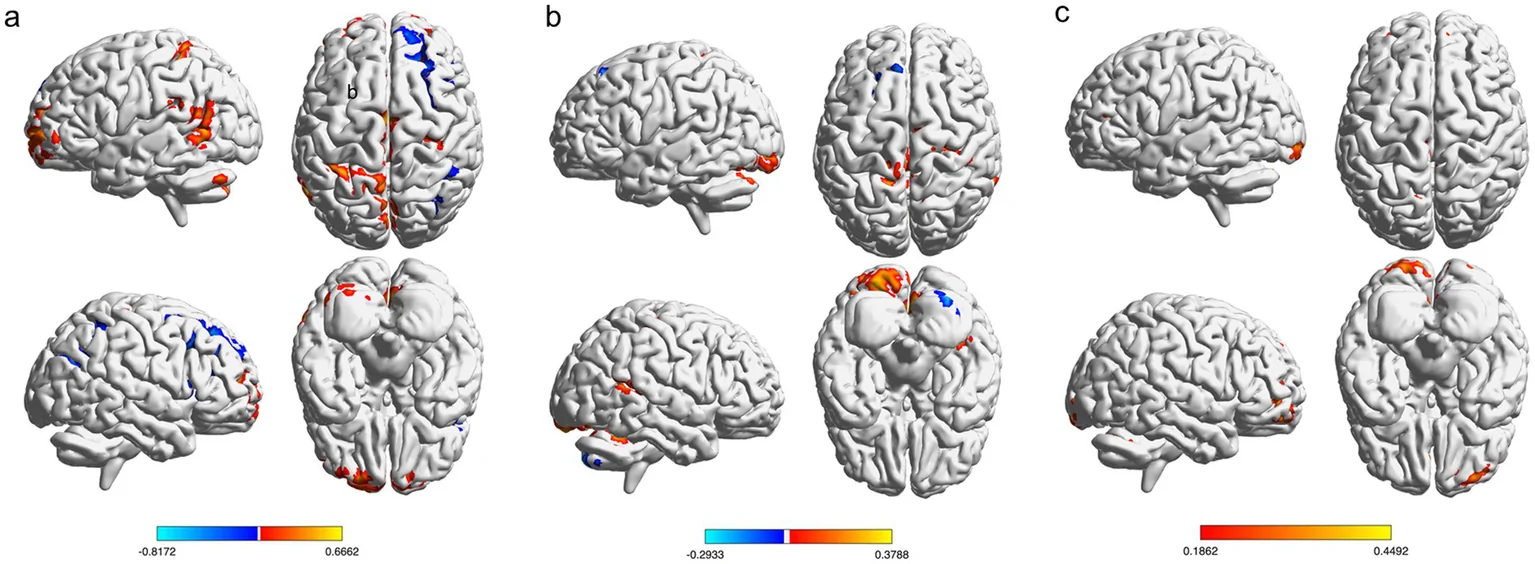
Postoperative changes in intrinsic functional connectivity after radiofrequency thermocoagulation. Seed-to-voxel resting-state functional connectivity (rs-fMRI) changes after RF-TC (postoperative minus preoperative) for the right frontal periventricular heterotopia (PVH) seed **(a)**, right centromedian nucleus (CM) seed **(b)**, and left CM seed **(c)**. Warm colors indicate increased connectivity and cool colors indicate decreased connectivity after treatment. The PVH seed showed decreased connectivity with ipsilateral frontal regions surrounding the treated territory, together with increased long-range connectivity involving posterior midline, supplementary motor area, and cerebellar regions. Bilateral CM seeds showed predominantly increased connectivity with widespread cortical and cerebellar territories. These rs-fMRI difference maps are descriptive measures of postoperative change in intrinsic functional connectivity and cannot support causal or directional inference.

### 3.5 Clinical outcome and patient perspective

At the 9-month follow-up, the patient remained seizure-free. Before RF-TC, disabling seizures caused falls approximately every 2 days and substantially restricted her daily activities. After RF-TC, these falls ceased, and the patient and her family reported improved physical safety, greater independence in daily activities, and reduced caregiver burden. The ASM regimen remained unchanged throughout follow-up. Given the sustained seizure freedom and functional improvement, the family was not considering surgical resection at the latest follow-up.

## 4 Discussion

### 4.1 Principal findings

This multimodal case provides direct invasive support for a focal lesion-driven secondary network model of LGS phenotype. SEEG identified the earliest sustained ictal activity within the sampled right frontal PVH-related medial contacts, accompanied by high-frequency activity. Ipsilateral lateral frontal recruitment preceded contralateral frontal synchronization, and CM activation followed cortical recruitment. In this patient, the CM therefore appeared to participate in secondary synchronization rather than seizure initiation. RF-TC of the driver and early propagation cortex produced seizure freedom. CCEP then showed selective weakening of lesion-driven effective output, whereas rs-fMRI demonstrated spatially specific postoperative changes in intrinsic connectivity, including increased PVH–CM coupling and decreased local frontal connectivity around the treated territory.

### 4.2 Comparison with previous SEEG-based and related network investigations

Previous SEEG studies have characterized LGS as a heterogeneous and distributed epileptogenic network involving bilateral cortical regions (Cho et al., 2025). Complementary EEG-fMRI studies have suggested that generalized paroxysmal activity may be cortically driven; for example, Warren et al. identified the prefrontal cortex as a primary driver within a cortical–brainstem–thalamic network (Warren et al., 2019). Our case extends these findings by linking the LGS phenotype to a structurally defined PVH-associated focal driver and demonstrating an ordered progression from focal onset to bilateral synchronization and subsequent CM recruitment. Furthermore, peri-interventional CCEP showed weakened lesion-associated effective output after RF-TC, while longitudinal rs-fMRI suggested accompanying intrinsic network changes. Together, these findings illustrate how a focal structural lesion may organize a secondary bilateral network underlying an apparently generalized LGS phenotype.

### 4.3 A focal network underlying generalized electroclinical features

These findings align with the concept of LGS as secondary network epilepsy, in which focal abnormalities recruit large-scale cortico-thalamic circuits (Archer et al., 2014; Warren et al., 2019). PVH-related epileptogenicity may extend beyond the nodule to adjacent and connected cortex (Tassi et al., 2004; Aghakhani et al., 2005). The presence of diffuse SSW, GPFA, multiple seizure types, and intellectual impairment established an LGS phenotype, but these scalp-level features did not identify the earliest intracranial node. SEEG demonstrated an ordered hierarchy from the medial right frontal network to ipsilateral lateral cortex, contralateral cortex, and bilateral CM. These observations suggest that generalized scalp EEG should not automatically preclude invasive focal evaluation when imaging, semiology, or other data support a plausible structural network substrate.

The transient seizure suppression during the first 4 days after implantation is consistent with a microlesional effect of electrode implantation (Kaur et al., 2019) and may have reduced the probability of capturing the full range of seizure semiologies documented on scalp EEG. This phenomenon may have reinforced the anatomical hypothesis guiding RF-TC (Bourdillon et al., 2018). Although this observation cannot localize the epileptogenic zone independently, it was concordant with the subsequent response to intervention within the sampled right frontal network.

### 4.4 Complementary information from CCEP and rs-fMRI

Postoperative CCEP findings provide physiological evidence that RF-TC weakened the effective output of the lesion-related network. Attenuation of lesion-centered and lesion-to-thalamus responses is consistent with a reduced capacity to recruit wider cortico-thalamic circuitry. Relative preservation of CM responses does not establish a primary thalamic role; instead, it is compatible with a persistent thalamocortical hub after interruption of cortical pathological output (Velasco et al., 1997; Warren et al., 2020).

Reduced CCEP responses after RF-TC and increased rs-fMRI correlations are therefore not inherently contradictory. CCEP measures stimulation-evoked propagation (Keller et al., 2014), whereas rs-fMRI indexes spontaneous BOLD synchrony (Centeno and Carmichael, 2014). Reduced local frontal functional connectivity around the PVH/RF-TC territory converged with reduced lesion-centered CCEP propagation. Increased long-range and CM-centered intrinsic connectivity may represent postoperative changes in residual thalamocortical networks after attenuation of pathological driving (Taylor et al., 2018; Morgan et al., 2020; Wang et al., 2026). Because the rs-fMRI PVH seed encompassed the entire malformation rather than only the SEEG-defined onset contacts, these findings should be interpreted as network-level associations, not evidence of directionality or causality.

### 4.5 Implications for CM-DBS in LGS-related networks

The findings also refine the rationale for CM-directed neuromodulation in LGS. CM-DBS may modulate a critical synchronizing node even when the CM is not the primary seizure generator (Valentín et al., 2013; Warren et al., 2020; Dalic et al., 2021). In the present recordings, CM recruitment followed bilateral frontal recruitment during seizures. This temporal order argues against the CM as the primary ictal generator in this individual, while remaining compatible with an important role in bilateral synchronization, seizure maintenance, or network amplification.

The relative preservation of responses between thalamic contacts after RF-TC further suggests that focal cortical treatment did not globally suppress thalamic responsiveness. This finding should not be interpreted as proof that anatomical intrathalamic connectivity was unchanged; CCEPs measure responses within the sampled electrode network under specific stimulation conditions. Nevertheless, the contrast between marked attenuation of right frontal lesion-related responses and stable bilateral thalamic responses supports a spatially selective intervention effect.

### 4.6 Clinical implications

This case supports individualized evaluation of LGS phenotype when a structural lesion such as PVH is present. Generalized scalp EEG and electroclinical features should not preclude SEEG-based focal surgical assessment, as a treatable lesion-related driver may be embedded within the apparent generalized epilepsy network. Multimodal connectivity findings should be interpreted in clinical and electrophysiological context. Although the CM remains an important therapeutic target for LGS-related networks, including CM-DBS, its contribution may vary across patients, supporting personalized network-guided treatment selection.

Beyond seizure control, the patient- and family-reported improvements in physical safety, daily independence, and caregiver burden indicate meaningful functional benefits of RF-TC. Although these outcomes were not assessed using standardized quality-of-life measures, they provide an important patient-centered perspective on treatment effectiveness.

### 4.7 Limitations

Several limitations should be acknowledged. This single-case report requires validation in larger cohorts of patients with LGS phenotypes and focal structural lesions. The SEEG sampling was hypothesis-driven and spatially limited; therefore, we cannot infer that the detected PVH-related activity represents the earliest generator in the entire brain, only the earliest detectable activity within the sampled regions. CCEP findings may be influenced by stimulation parameters, electrode coverage, antiseizure medications, and postoperative tissue changes. The rs-fMRI findings are correlational and exploratory, and RF-TC–related lesioning within the PVH seed may have influenced the observed connectivity changes. In addition, SEEG contact localization and CM segmentation depend on image registration and atlas-based methods. Finally, postoperative changes may reflect the combined effects of RF-TC, seizure reduction, and developmental or state-dependent factors. Despite these limitations, the convergence of SEEG, CCEP, and rs-fMRI findings supports a coherent model of focal lesion-driven secondary network epilepsy.

## 5 Conclusion

In this patient, a right frontal PVH-related epileptogenic network was associated with an LGS phenotype through secondary bilateral cortical and delayed CM recruitment. After SEEG-guided RF-TC, seizure freedom was achieved; CCEP demonstrated attenuation of lesion-driven effective propagation; and postoperative rs-fMRI revealed spatially patterned longitudinal changes in intrinsic functional connectivity. Multimodal assessment may help identify focal drivers versus propagation and synchronizing nodes in secondary network epilepsies.

## Data Availability

The original contributions presented in the study are included in the article/Supplementary material, further inquiries can be directed to the corresponding author.

## References

[ref1] Aghakhani Y. Kinay D. Gotman J. Soualmi L. Andermann F. Olivier A. . (2005). The role of periventricular nodular heterotopia in epileptogenesis. Brain 128, 641–651. doi: 10.1093/brain/awh388, 15659421

[ref2] Archer J. S. Warren A. E. L. Stagnitti M. R. Masterton R. A. J. Abbott D. F. Jackson G. D. (2014). Lennox-Gastaut syndrome and phenotype: secondary network epilepsies. Epilepsia 55, 1245–1254. doi: 10.1111/epi.12682, 24902608

[ref3] Arzimanoglou A. French J. Blume W. T. Cross J. H. Ernst J.-P. Feucht M. . (2009). Lennox-Gastaut syndrome: a consensus approach on diagnosis, assessment, management, and trial methodology. Lancet Neurol. 8, 82–93. doi: 10.1016/s1474-4422(08)70292-8, 19081517

[ref4] Bourdillon P. Cucherat M. Isnard J. Ostrowsky-Coste K. Catenoix H. Guénot M. . (2018). Stereo-electroencephalography-guided radiofrequency thermocoagulation in patients with focal epilepsy: a systematic review and meta-analysis. Epilepsia 59, 2296–2304. doi: 10.1111/epi.14584 30345535

[ref5] Centeno M. Carmichael D. W. (2014). Network connectivity in epilepsy: resting state fMRI and EEG-fMRI contributions. Front. Neurol. 5:93. doi: 10.3389/fneur.2014.00093 PMC4081640 25071695

[ref6] Cho S. Makhalova J. Medina Villalon S. Villeneuve N. Trébuchon A. Krouma M. . (2025). Stereoelectroencephalographic exploration and surgical outcome in Lennox–Gastaut syndrome. Epilepsia 66, 1417–1432. doi: 10.1111/epi.18283, 39871521 PMC12097472

[ref7] Dalic L. J. Warren A. E. L. Bulluss K. J. Thevathasan W. Roten A. Churilov L. . (2021). DBS of thalamic centromedian nucleus for Lennox-Gastaut syndrome (ESTEL trial). Ann. Neurol. 91, 253–267. doi: 10.1002/ana.26280 34877694

[ref8] Kaur M. Szaflarski J. P. Ver Hoef L. Pati S. Riley K. O. Jaisani Z. (2019). Long-term seizure freedom following intracranial sEEG monitoring: therapeutic benefit of a diagnostic technique. Epilepsy Behav. Rep. 12:100345. doi: 10.1016/j.ebr.2019.100345, 31799508 PMC6883308

[ref9] Keller C. J. Honey C. J. Mégevand P. Entz L. Ulbert I. Mehta A. D. (2014). Mapping human brain networks with cortico-cortical evoked potentials. Philos. Trans. R. Soc. B Biol. Sci. 369:20130528. doi: 10.1098/rstb.2013.0528, 25180306 PMC4150303

[ref10] Morgan V. L. Rogers B. P. Anderson A. W. Landman B. A. Englot D. J. (2020). Divergent network properties that predict early surgical failure versus late recurrence in temporal lobe epilepsy. J. Neurosurg. 132, 1324–1333. doi: 10.3171/2019.1.jns182875, 30952126 PMC6778487

[ref11] Specchio N. Wirrell E. C. Scheffer I. E. Nabbout R. Riney K. Samia P. . (2022). International league against epilepsy classification and definition of epilepsy syndromes with onset in childhood: position paper by the ILAE task force on nosology and definitions. Epilepsia 63, 1398–1442. doi: 10.1111/epi.17241, 35503717

[ref12] Tassi L. Colombo N. Cossu M. Mai R. Francione S. Lo Russo G. . (2004). Electroclinical, MRI and neuropathological study of 10 patients with nodular heterotopia, with surgical outcomes. Brain 128, 321–337. doi: 10.1093/brain/awh357, 15618282

[ref13] Taylor P. N. Sinha N. Wang Y. Vos S. B. de Tisi J. Miserocchi A. . (2018). The impact of epilepsy surgery on the structural connectome and its relation to outcome. Neuroimage Clin. 18, 202–214. doi: 10.1016/j.nicl.2018.01.028, 29876245 PMC5987798

[ref14] Valentín A. García Navarrete E. Chelvarajah R. Torres C. Navas M. Vico L. . (2013). Deep brain stimulation of the centromedian thalamic nucleus for the treatment of generalized and frontal epilepsies. Epilepsia 54, 1823–1833. doi: 10.1111/epi.12352, 24032641

[ref15] Velasco M. Velasco F. Velasco A. L. Brito F. Jiménez F. Marquez I. . (1997). Electrocortical and behavioral responses produced by acute electrical stimulation of the human centromedian thalamic nucleus. Electroencephalogr. Clin. Neurophysiol. 102, 461–471. doi: 10.1016/s0013-4694(96)95203-0, 9216479

[ref16] Wang C. Chen B. Hong J. Xu J. (2026). Network reprogramming after resection of occipital meningioangiomatosis: evidence from multimodal localization and longitudinal fMRI. Epilepsy Behav. Rep. 34:100874. doi: 10.1016/j.ebr.2026.100874 PMC13266237 42306261

[ref17] Warren A. E. L. Dalic L. J. Thevathasan W. Roten A. Bulluss K. J. Archer J. (2020). Targeting the centromedian thalamic nucleus for deep brain stimulation. J. Neurol. Neurosurg. Psychiatry 91, 339–349. doi: 10.1136/jnnp-2019-322030, 31980515

[ref18] Warren A. E. L. Harvey A. S. Vogrin S. J. Bailey C. Davidson A. Jackson G. D. . (2019). The epileptic network of Lennox-Gastaut syndrome: cortically driven and reproducible across age. Neurology 93:e215–e226. doi: 10.1212/WNL.0000000000007775 31227617

