# Supplementary material for: Case Report: Right frontal periventricular heterotopia underlying secondary network epilepsy with a Lennox–Gastaut phenotype: multimodal SEEG, CCEP, and rs-fMRI evidence
Source: Front Neurosci. 2026 Sep 30;20:1961911. doi: 10.3389/fnins.2026.1961911 (PMC13661969; doi:10.3389/fnins.2026.1961911)
Supplement: Supplementary file 1 [file Supplementary_file_1.docx]

(a)

(b)

(c)

(d)

(e)

(f)

(g)

**Supplementary Figure S1.** Representative scalp EEG and multimodal structural imaging supporting an LGS phenotype.

(a) Interictal scalp EEG showing generalized slow spike-and-wave discharges at approximately 1.5–2.5 Hz.

(b) Ictal scalp EEG during a tonic seizure showing generalized fast activity change.

Recording parameters are indicated on the left of each panel (sensitivity, high-frequency filter, low-frequency filter, and calibration).

(c–g) Multiplanar structural imaging (MRI and PET) including two T1 sequences, FLAIR, T2, and PET, shown in axial, coronal, and sagittal planes. The periventricular gray-matter abnormality is emphasized by its gray-matter protrusion toward the ventricle, and the gray–white matter interface is illustrated with clear delineation.

(a)

(b)

 **Supplementary Figure S2.** Second ictal SEEG pattern.

(a) Lateral frontal slow-wave/sharp-wave activity preceded voltage attenuation and high-frequency recruitment. However, high-frequency activity emerged earlier in medial lesion-related contacts than in lateral frontal contacts. Contralateral frontal (A’) and bilateral centromedian (R, L’) involvement followed cortical recruitment (b). A-E, right frontal lesion-related and perilesional contacts; R, right centromedian nucleus; A’, contralateral frontal cortex; and L’, left centromedian nucleus.

**Supplementary Figure S3.** CCEP responses to left-sided stimulation before and after RF-TC. Heatmaps show CCEP responses before (A) and after (B) RF-TC. Rows show all left-sided bipolar stimulation pairs, and columns show response channels from both hemispheres. Response strength was quantified as the RMS amplitude from 15 to 300 ms after stimulus onset. Crossed gray cells indicate contacts corresponding to the stimulated pair and were excluded from the response analysis. Darker blue indicates a stronger response.

**Supplementary Table S1.** Summary of SEEG ictal categories, number of seizures, onset sub-patterns, and associated medial PVH-related core onset (HFA), together with propagation hierarchy and event reproducibility.

| SEEG ictal category | Number of seizures | Onset sub-patterns | Medial PVH-related core onset (HFA) | Propagation hierarchy to ipsilateral frontal → contralateral frontal → bilateral CM | Reproducibility |
| --- | --- | --- | --- | --- | --- |
| Spasm–tonic (ST) | 10 | 2 (sleep-related vs wake-related) | Yes | Yes (consistent order) | Yes (all 10) |
| ST → FBTCS | 1 | 1 | Yes | Yes (+ secondary generalization) | Yes (single event) |

ST denotes spasm–tonic seizures; ST → FBTCS indicates evolution from ST onset to FBTCS. Onset sub-patterns are further stratified by sleep-related vs wake-related initiation. Medial PVH-related core onset (HFA) indicates the presence of an HFA core onset involving the medial PVH region. Propagation hierarchy to ipsilateral frontal → contralateral frontal → bilateral CM describes the observed stepwise spread pattern across these regions. Reproducibility reflects the consistency of the onset and propagation order across seizures of the same ictal category.

**Supplementary Table S2.** Preoperative and postoperative ROI‑to‑ROI functional connectivity (zFC) and postoperative change.

| **Connection** | **Pre zFC** | **Post zFC** | **ΔzFC** |
| --- | --- | --- | --- |
| ROI1–ROI2 | 0.2099 | 0.2633 | 0.0534 |
| ROI1–ROI3 | 0.1065 | 0.2594 | 0.1529 |
| ROI2–ROI3 | 0.2799 | 0.3445 | 0.0645 |

ROI1, entire right frontal periventricular heterotopia lesion (manually delineated); ROI2, right centromedian thalamic nucleus; ROI3, left centromedian thalamic nucleus. zFC, Fisher’s z-transformed correlation coefficient; Δ, postoperative minus preoperative.

ROI-to-ROI rs-fMRI analysis showed increased postoperative Fisher z-transformed connectivity among the lesion-related cortical/PVH region, bilateral CM, and inter-CM connections.

Postoperative zFC increased between the cortical seeds and bilateral CM: ROI1–ROI2 from 0.2099 to 0.2633 (Δ=+0.0534), and ROI1–ROI3 from 0.1065 to 0.2594 (Δ=+0.1529). Inter‑thalamic connectivity (ROI2–ROI3) also increased from 0.2799 to 0.3445 (Δ=+0.0645).
